# Five Cases of Extra-Uterine Pregnancy, Operated upon at the Time of Rupture

**Published:** 1884-11

**Authors:** 


### Five Cases of Extra-Uterine Pregnancy, Operated Upon at the Time of Rupture.

Several weeks since, Mr. Lawson Tait reported, in the *British Medical Journal* a series of five cases of extra-uterine pregnancy, operated upon at the time of rupture, from the tenth to the thirteenth week. Four were successful. In each he removed the fallopian tube, the pregnancy having been tubal. In his own words: "These cases all confirm the view of the pathology of extra-uterine pregnancy which I advanced many years ago, that in origin it is always tubal, and that its varieties depend merely upon the direction in which rupture occurs. These results also confirm the policy of early interfering in such cases, for four out of the five have been easily and completely cured of one of the most formidable conditions of pregnancy. The first and only fatal case might have had a better ending if Mr. Spachman had seen her sooner, for he recognized the case at once and sent for me."

